# Sprout Caffeoylquinic Acid Profiles as Affected by Variety, Cooking, and Storage

**DOI:** 10.3389/fnut.2021.748001

**Published:** 2021-12-13

**Authors:** Gholamreza Khaksar, Ketthida Cheevarungnapakul, Patwira Boonjing, Supaart Sirikantaramas

**Affiliations:** ^1^Molecular Crop Research Unit, Department of Biochemistry, Faculty of Science, Chulalongkorn University, Bangkok, Thailand; ^2^Program in Biotechnology, Faculty of Science, Chulalongkorn University, Bangkok, Thailand; ^3^Omics Sciences and Bioinformatics Center, Chulalongkorn University, Bangkok, Thailand

**Keywords:** bioactive compounds, caffeoylquinic acids, cooking, low temperature storage, sunflower sprout, variety

## Abstract

Various health-promoting properties inherent to plant-based foods have been attributed to their rich bioactive compounds, including caffeoylquinic acids (CQAs). The potential health benefits of CQAs have been well-documented. While sprouts are widely recognized as health-promoting foods owing to their high phytonutrient content, our knowledge regarding the effect of cooking and storage, commonly practiced by consumers, on the CQA content remains limited. First, sunflower sprouts were found to have the highest total CQA content (~ 22 mg/g dry weight) out of 11 commonly available sprouts. Then, the effect of variety, cooking, and low-temperature storage on the CQA profile of sunflower sprouts was investigated. Among the four different varieties of sunflower sprouts, variety 1 harbored the highest total CQA content. Notably, cooking adversely affected the CQA content of sunflower sprouts relative to the uncooked samples in a time-dependent manner, possibly due to the heat sensitivity of CQAs. Under simulated home-refrigeration storage conditions, we observed a significant decline in the content of major CQA compounds (5-monoCQA and 3,5-diCQA) at days 10 and 13 of storage. The results obtained herein provide consumers and food industrialists with increased insight into the effect of cooking and refrigeration on the CQA content of sunflower sprouts.

## Introduction

Lifestyle changes and increased awareness among health-conscious consumers have led to an increasing interest in healthy diets of natural origin. There is a large body of evidence indicating a strong association between high plant product intake and reduced risk factors related to major chronic diseases in humans (1), including cardiovascular disease (CVD) (2), cancer (3), and age-related degeneration (4). The health-promoting properties of plants are attributed to the rich content of bioactive compounds, such as vitamins C and E, carotenoids, and phenolic compounds, that possess antioxidant activity (5).

Phenolic compounds exist in plants in various forms, including aglycones (free phenolic acids), esters, glycosides, and bound complexes (6). Among these, caffeoylquinic acids (CQAs) are characterized as esters of hydroxycinnamic acids and quinic acid, which are divided into three main groups: monocaffeoylquinic acids (monoCQAs with at least three isoforms: 3-CQA, 4-CQA, and 5-CQA, known as chlorogenic acid), dicaffeoylquinic acids (diCQAs with at least three isoforms: 3,4-diCQA, 3,5-diCQA, and 4,5-diCQA) (7–9), and tricaffeoylquinic acid (triCQA with one isoform: 3,4,5-triCQA) (10). Owing to their structural diversity, CQAs exert a wide range of biological activities. CQAs have numerous health-promoting characteristics, such as antimicrobial (antibacterial and antiviral), antioxidant, and anti-inflammatory properties, which can substantially reduce the rates of chronic and CVDs, as described by several *in vivo* and *in vitro* studies (11–14). In addition, CQAs interfere with the absorption and biosynthesis of cholesterol and contribute to low-density lipoprotein (LDL) reduction (15). Notably, 5-CQA [chlorogenic acid (CGA)] modifies glucose metabolism, which benefits the treatment of injuries caused by diabetes (16) and protects endothelial cells from oxidative stress (17). They can also inhibit HIV replication and integration (18). In plants, CQAs can enhance resistance to biotic (19, 20) and abiotic (21) stressors by acting as antioxidants and scavenging free radicals.

Seed germination is a complex stage of plant ontogenesis that activates the seed embryo and allows for seedling growth (22, 23). Seven- or 10-day-old sprouts are ideal for harvesting, allowing for post-harvest handling and commercialization (23). Notably, sprouts have a higher content of phytochemicals than other vegetables (23). Health promotion guidelines and dietary recommendations widely recommend regular consumption of sprouts due to their high content of nutrients and bioactive compounds, including CQAs (24, 25). In view of the ever-increasing interest in healthy lifestyles and prevention of diseases, sprouts are highly desirable products of plant origin. Apart from their health-promoting properties, sprouts can be easily and quickly produced and are considered innovative culinary ingredients of high popularity due to their delicate texture, unique colors, and high palatability. Various species can be consumed as sprouts, some of which are of great popularity, such as cereals, legumes, oilseeds, and crucifers, which include lentils, soybean, broccoli, alfalfa, radish, mung bean, and sunflower.

Sunflowers (*Helianthus annuus* L.) belong to the Asteraceae family and are grown commercially worldwide. Sunflower seeds are utilized as an important source of vegetable oil worldwide (26). Sunflower seeds and sprouts have health-promoting properties, including antimicrobial, antioxidant, anti-inflammatory, antihypertensive, wound-healing, and cardiovascular benefits, due to its bioactive compounds that include phenolic compounds, polyunsaturated fatty acids, and vitamins (27). The notable nutritional, medicinal, and culinary benefits of sunflower seeds and sprouts have resulted in an ever-growing popularity among health-conscious consumers worldwide. Sun et al. (28) investigated the antiglycative and antioxidant characteristics of four edible sprouts and found that sunflower sprout extract had antiglycative properties similar to those of aminoguanidine, a well-known synthetic antiglycative agent. Moreover, the strong antioxidant and antiglycative capacity of sunflower sprouts was found in its rich diCQA content. Consistently, Cheevarungnapakul et al. (29) profiled the CQA content in sunflower sprouts and found both mono and diCQAs. Notably, seed germination affects the content of CQAs in sunflowers. Paja̧k et al. (30) investigated the effect of germination on bioactive compound content (total phenolics and flavonoids) and antioxidant properties of sunflower seeds. Total phenolics, flavonoids, and antioxidant capabilities were significantly higher in sunflower sprouts than in seeds. Moreover, the CQA content showed a 3.7-fold increase in sprouts compared with seeds (30).

Storing sprouts in a refrigerator at home and consuming them as desired is a regular practice. Sprouts are used in various ways. They can be used raw as additions to sandwiches, salads, soups, and desserts, and/or they can be cooked. Thus, it is of paramount importance for consumers and food industries to understand the effect of low-temperature storage and cooking on the content of bioactive compounds in sprouts. Previous studies have documented the effect of cold storage on the phytonutrient content and bioactive properties of various fruits and vegetables, suggesting different patterns of variation in the bioactive compound content under simulated home-refrigeration storage conditions, depending on the species and compounds being investigated (31–39). However, our knowledge regarding the effect of low-temperature storage and cooking on the phytonutrient content of sprouts remains poorly understood and limited to only a few studies.

Swieca and Gawlik-Dziki (40) investigated the effect of low-temperature storage on phenolic content, antioxidant activity, and starch content in green pea, lentil, and mung bean sprouts. It was found that the bioactivity and nutritional quality of sprouts were affected by storage at low temperatures. In addition, storage significantly enhanced the starch digestibility and glycemic index value, which was linked to the reduction of resistant starch content. Notably, sprouts are usually consumed after cooking, which can be performed in different ways, according to personal preferences and/or culinary traditions. However, steaming is considered as one of the most common methods of cooking (41, 42). In addition, steaming has been proved to maintain or increase the total phenolic content in some vegetables (43). Hwang (42) investigated the effect of different cooking methods on the total bioactive compound content of Brussels sprouts. Microwaved Brussels sprouts contained the highest amount of total carotenoids and chlorophylls, followed by steamed and uncooked samples. However, microwaving and steaming deteriorated the antioxidant activity and total flavonoid content of Brussels sprouts. In another study by Chiavaro et al. (44), *sous vide* processed Brussels sprouts contained higher carotenoid content but lower phenolic compounds, ascorbic acid, and antioxidant activity when compared to those of steamed samples. Kumari and Chang (45) demonstrated that cooking caused significant losses to the antioxidant capacity and phenolic content of soy sprouts.

Of particular note, different varieties of one sprout species can harbor different health-promoting properties, likely due to the different contents of phytochemicals, which can be attributed to climatic conditions of sprout cultivation and/or different genotypes. Limmongkon et al. (46) reported a significant difference in phenolic content and antioxidant capacity among the five varieties of peanut sprouts.

Herein, considering the significant nutritional and culinary benefits of sprouts, we profiled the CQA content of various sprout cultivars commonly found in the market in Thailand and found that sunflower sprouts had the highest CQA content. We then examined the effect of low-temperature storage, cooking, and variety on the CQA content of sunflower sprouts. To our knowledge, this is the first report on the effect of these factors on the CQA content of sunflower sprouts. The results obtained from this study will increase consumer awareness regarding the effect of refrigerated storage and cooking on the nutritional value of sunflower sprouts.

## Materials and Methods

### Chemicals

The commercial standards of CQAs (monoCQAs: 1-CQA, 3-CQA, 4-CQA, and 5-CQA and diCQAs: 1,3-diCQA, 1,4-diCQA, 1,5-diCQA, 3,4-diCQA, and 4,5-diCQA) were purchased from Biosynth Carbosynth^®^, UK (purity ≥ 98.0%), and 3,5-diCQA was purchased from TransMIT GmbH PlantMetaChem (Germany). Puerarin (internal standard) was obtained from Sigma Aldrich (St. Louis, MO, USA; purity ≥ 98.0%). Acetonitrile and ethanol (high performance liquid chromatography (HPLC) grade) were obtained from Merck (Darmstadt, Germany). Ultra-pure water used for all experiments was obtained from a Milli-Q system (Millipore, Billerica, MA, USA).

### Plant Materials and Seed Germination

The sprouts of broccoli (*Brassica oleracea* var. *italica*), Chinese kale (*Brassica oleracea* Alboglabra Group), green pea (*Pisum sativum*), mung bean (*Phaseolus vulgaris*), mustard (*Brassica nigra*), pea (*Pisum sativum*), peanut (*Arachis hypogaea*), purple cabbage (*Brassica oleracea* var. *Capitata* f. *rubra*), soybean (*Glycine max*), sunflower (*Helianthus annuus* L.), and white radish (*Raphanus sativus*) were purchased from a local supermarket in Bangkok, Thailand (pictures of representative sprout species are presented in Supplementary Figure 1). Three independent biological replicates were used for each sprout species. The sprout samples were washed with water and freeze-dried. The CQA profiles of each sprout species were analyzed. The species with the highest total CQA content (sunflower sprouts) was selected for further study.

To investigate the effect of variety on CQA content, seeds of four different varieties of sunflower sprouts (1: Lungtop line, 2: Chiatai, 3: 3A, 4: Fourtid) were purchased from a local supermarket in Bangkok, Thailand (pictures of representative seeds and sprouts are shown in Supplementary Figure 2). These four varieties are commonly available in Thai local seed shops. The seeds were then washed with tap water, soaked for 8 h, and wrapped with wet cheesecloth overnight. Seeds were then germinated on coconut dust under controlled conditions (temperature 30°C and 60% relative humidity) for 5 days, under dark conditions for the first 48 h, followed by a 12/12 h-light/dark photoperiod for the remaining days (29).

### Cooking Treatment and Storage at 4°C

We used steaming to investigate the effect of cooking on the CQA profile of sunflower sprouts relative to the raw (uncooked) samples. We also varied the cooking period (2, 5, 7, and 10 min) on the knowledge of different cooking habits among chefs. For each cooking period, sunflower sprouts were weighed, washed, and dried with tissue paper. After that, the sprouts were cooked (steamed) using an electric food steamer. Following cooking, the samples were immediately frozen in liquid nitrogen and freeze-dried with the application of Gamma 1-16 LSC freeze-dryer at a shelf temperature of 20°C, ice condenser temperature of −55°C and pressure of 63 Pa for 24 h. To investigate the effect of storage conditions (time-period) on the CQA content of sunflower sprouts, sprouts were stored at 4°C (simulated home-refrigerated storage) and sampled at day 1, 2, 3, 5, 7, 10, and 13 of storage.

### Extraction of CQAs From Sprout Samples

Methanolic extracts were prepared to quantify the CQA content of the sprout samples. To this end, freeze-dried samples were ground to a fine powder using a mixer mill (MM 400, Retsch, Germany) at 30 Hz for 1 min. After that, 20 mg (dry weight) of each ground sample was extracted with 1 mL aqueous solution of 80% (v/v) methanol containing an internal standard, puerarin (0.05 g L^−1^) by shaking vigorously at 1,500 rpm for 15 min at 15°C using an Eppendorf Thermomixer® C (Eppendorf, USA). The mixtures were then centrifuged at 12,000 × *g* for 15 min at 4°C using a table-top 5415 R centrifuge machine (Eppendorf, USA). Supernatants were collected, filtered through a 0.2-μm nylon syringe filter, and injected into a high-performance liquid chromatograph to analyze the CQAs (29).

### HPLC

CQA profiling of the sprout samples was performed using an UltiMate 3000 HPLC liquid chromatography system coupled with a Dionex UltiMate DAD 3000 detector (Thermo Fisher Scientific, Waltham, MA, USA) with a Kinetex EVO C18 (250 mm × 4.6 mm, 5 μm) (Phenomenex, USA) using UV at 320 nm following the method described by Cheevarungnapakul et al. (29) with minor modifications. The mobile phase was composed of water with 0.1% (v/v) formic acid (TFA) (pH 2.4; eluent A) and acetonitrile with 0.1% (v/v) TFA (eluent B) with the following gradient program: 5% B (20 min), 5–15% B (10 min), 15% B (25 min), a 4-min hold, 100% B (3 min), a 4-min hold, and 5% B (5 min). The flow rate was 1.5 mL min^−1^ with an injection volume of 10 μL. Peaks corresponding to the retention time and UV spectrum of a commercial standard (standards used in this study were mentioned in section Chemicals) were identified as CQAs. Quantification of each CQA was performed according to the calibration curve in the range of 1.30–500 μg/mL. Puerarin was used as an internal standard. Supplementary Figure 3 presents the chromatograms of the analytical standards and the sunflower sprout sample. More information regarding the standards used in this study, including standard curves, equations, limit of detection (LOD), and limit of quantification (LOQ) are presented in Supplementary Figure 4 and Supplementary Table 1.

### Statistical Analysis

Data were subjected to statistical analysis using SPSS software, version 20 (SPSS Inc., IBM). Data are presented as the mean ± standard deviation (SD) of three independent replicates. Statistical comparisons of the means were performed using a one-way ANOVA, followed by Duncan's multiple range test (variety and cooking experiments) or Student's *t*-test (4°C-storage experiment) at a confidence level of 0.05.

## Results

### CQA Profiling of Different Sprout Species

Using HPLC, we profiled the CQA content of 11 different sprouts commonly found in Thai local markets. A total of nine CQA compounds, including monoCQA (1-, 3-, 5-, and 4-CQA) and diCQA (1,3-, 3,4-, 1,5-, 3,5-, and 4,5-diCQA) were identified and quantified (Table 1). Among the sprouts, sunflower sprouts harbored the highest content of both monoCQA and diCQA (22.01 ± 1.07 mg/g), followed by broccoli (15.32 ± 1.22 mg/g), green pea (12.3 ± 0.51 mg/g), and Chinese kale (11.22 ± 0.87) sprouts on dry weight basis (mg/g dry weight) (Table 1). However, pea (4.41 ± 0.19 mg/g dry weight) and purple cabbage (3.99 ± 0.55 mg/g dry weight) sprouts contained the lowest total CQA content (Table 1). Considering the highest total CQA content among the sprout species, sunflower sprouts were selected as candidate sprouts for our study.

**Table 1:** CQA profiles and contents of 11 sprouts.

**Common name**	**Scientific name**	**1-CQA**	**3-CQA**	**5-CQA**	**4-CQA**	**1,3-CQA**	**3,4-CQA**	**1,5-CQA**	**3,5-CQA**	**4,5-CQA**	**Total CQA**
Sunflower	*Helianthus annuus*	nd	0.95 ± 0.06	6.7 ± 0.78	0.13 ± 0.008	nd	0.55 ± 0.05	nd	12.87 ± 1.08	0.55 ± 0.11	22.01 ± 1.07^a^
Broccoli	*Brassica oleracea* var. *italica*	0.08 ± 0.005	0.76 ± 0.09	4.98 ± 0.87	0.09 ± 0.007	nd	0.33 ± 0.09	0.09 ± 0.005	6.75 ± 0.98	1.55 ± 0.11	15.32 ± 1.22^b^
Green pea	*Pisum sativum*	0.09 ± 0.003	1.65 ± 0.13	3.32 ± 0.77	nd	nd	0.21 ± 0.09	0.11 ± 0.08	4.55 ± 0.66	2.87 ± 0.55	12.3 ± 0.51^c^
Chinese kale	*Brassica oleracea* Alboglabra group	nd	0.43 ± 0.008	3.43 ± 0.009	nd	nd	nd	nd	5.84 ± 0.32	1.08 ± 0.09	11.22 ± 0.87^c^
White radish	*Raphanus sativus*	nd	0.46 ± 0.07	2.98 ± 0.09	nd	nd	0.14 ± 0.08	nd	4.09 ± 0.41	2.88 ± 0.21	10.88 ± 0.09^d^
Mung bean	*Phaseolus vulgaris*	0.07 ± 0.002	0.21 ± 0.08	4.88 ± 0.42	nd	nd	0.08 ± 0.01	nd	3.44 ± 0.32	0.22 ± 0.07	9.54 ± 0.44^e^
Soybean	*Glycine max*	nd	nd	1.08 ± 0.08	nd	nd	0.11 ± 0.09	nd	4.98 ± 0.09	1.98 ± 0.11	8.08 ± 0.33^f^
Mustard	*Brassica nigra*	nd	nd	3.11 ± 0.86	0.09 ± 0.008	0.08 ± 0.006	0.23 ± 0.08	nd	4.21 ± 0.54	0.21 ± 0.09	7.99 ± 0.07^f^
Peanut	*Arachis hypogaea*	nd	0.33 ± 0.03	3.44 ± 0.33	nd	nd	nd	nd	2.77 ± 0.11	1.11 ± 0.33	7.98 ± 0.09^f^
Pea	*Pisum sativum*	nd	nd	2.31 ± 0.08	nd	nd	0.11 ± 0.06	nd	2.07 ± 0.08	0.08 ± 0.008	4.41 ± 0.19^g^
Purple cabbage	*Brassica oleracea* var. *Capitata* f. *rubra*	nd	nd	2.09 ± 0.08	nd	nd	0.11 ± 0.08	nd	1.09 ± 0.07	0.87 ± 0.11	3.99 ± 0.55^g^

*Data are presented as the mean ± standard deviation (SD) of three independent replicates, expressed in mg/g of dry weight. For total CQA content, values followed by different superscript letters are significantly different according to Duncan's multiple-range test (p < 0.05). nd, not detected. 1,4-diCQA was not detected in any of the studied samples. Therefore, it is not included*.

### CQA Profile of Different Sunflower Sprout Varieties

**Figure 1:**
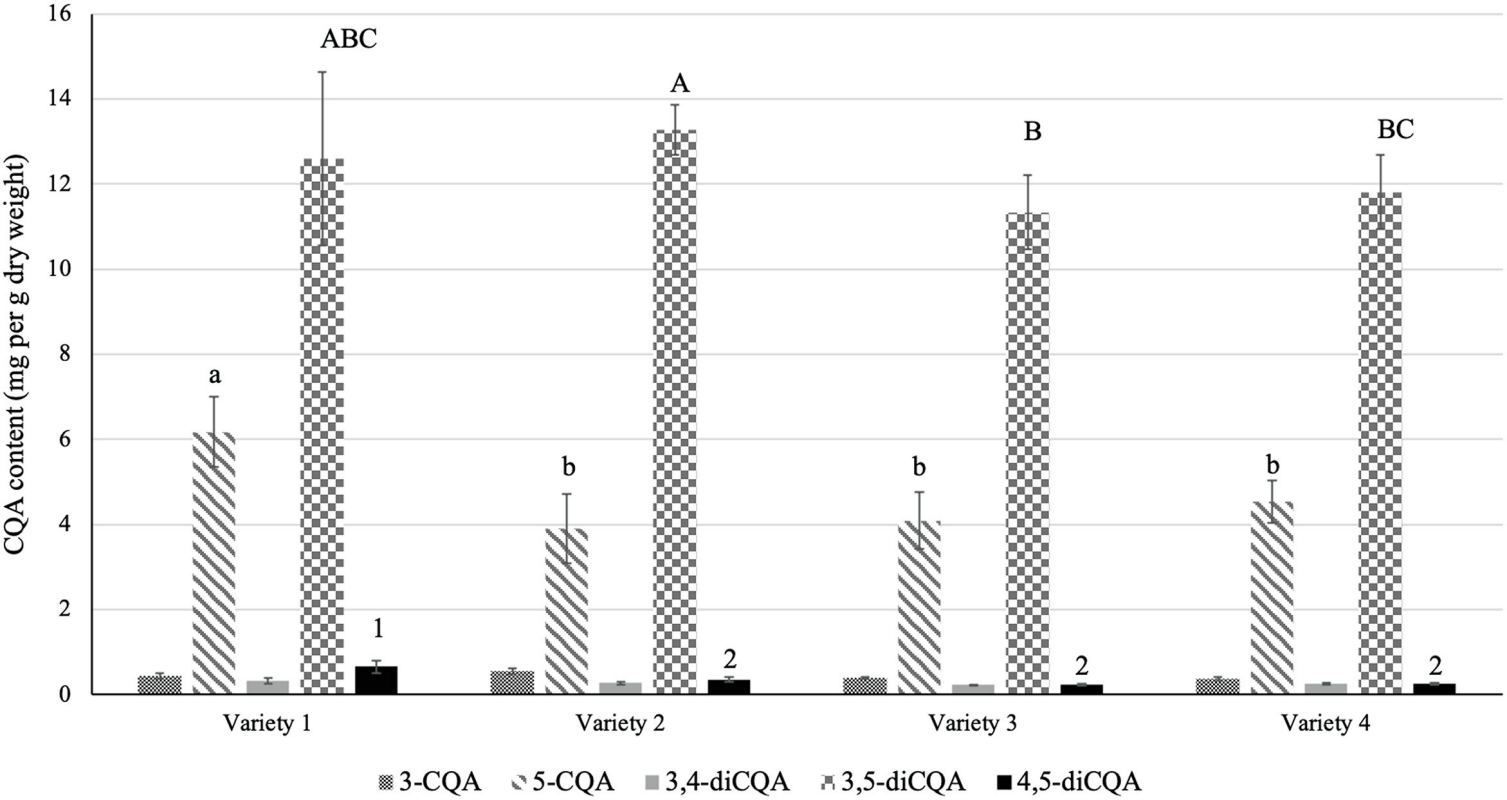
CQA content of different sunflower sprout varieties. Seeds of four different varieties of sunflower sprout (1: Lungtop line, 2: Chiatai, 3: 3A, 4: Fourtid) were purchased and germinated. Sprouts were then extracted for CQA profiling. Error bars represent means ± standard deviations (SD), and three independent biological replicates were performed. For each CQA derivative, comparisons are made among varieties; bars with different letters or numbers differed significantly according to Duncan's multiple range test (*P* < 0.05). The content of 3-CQA and 3,4-diCQA did not vary significantly among the varieties according to Duncan's multiple range test (*P* > 0.05).

We profiled the CQA content of different sunflower sprout varieties from commercialized seeds in Thailand to investigate the effect of variety on CQA content. HPLC profiling identified and quantified a total of five CQA compounds in the sprouts, including monoCQAs (3-CQA and 5-CQA) and diCQAs (3,4-diCQA, 3,5-diCQA, and 4,5-diCQA; Figure 1). Among the samples, variety 1 harbored the highest total CQA content (21.02 ± 0.75 mg/g) followed by variety 2 (18.55 ± 0.31 mg/g) on dry weight basis, and the total CQA content did not differ significantly between the other two varieties. The contribution of each of these two classes (mono and diCQA) to the total CQA content did not vary according to the variety. As presented in Figure 1, the contribution of diCQA isomers to the total CQA content was predominant. Notably, for monoCQA, 5-CQA was the major isomer in all samples, whereas for diCQA, 3,5-diCQA was the most abundant (Figure 1).

### Effect of Cooking on the CQA Content of Sunflower Sprouts

**Figure 2:**
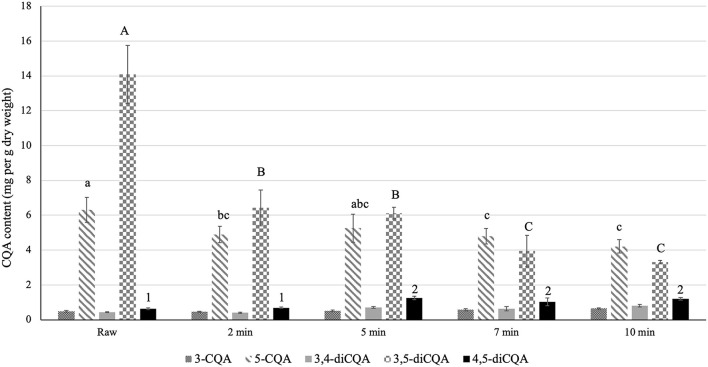
Effect of cooking on the CQA content of sunflower sprouts. The CQA profile of sunflower sprouts were analyzed after cooking under controlled conditions (steaming for different time periods, including 2, 5, 7, and 10 min). Error bars represent means ± standard deviations (SD), and three independent biological replicates were performed. For each CQA derivative, comparisons were made among uncooked (raw) and cooked samples at different timepoints; bars with different letters or numbers differed significantly according to Duncan's multiple range test (*P* < 0.05). The content of 3-CQA and 3,4-diCQA did not vary significantly among raw and cooked samples according to Duncan's multiple range test (*P* > 0.05).

Consuming vegetables after cooking is a common practice among consumers. Accordingly, to investigate the possible effect of cooking on the CQA content of sunflower sprouts, we analyzed the CQA profile after controlled cooking using an electric food steamer for different time periods, including 2, 5, 7, and 10 min. Notably, cooking detrimentally affected the total CQA content of sunflower sprouts relative to the raw (uncooked) sprouts in a time-dependent manner (Figure 2). The total CQA content reached its lowest value at 7 and 10 min of cooking (Figure 2). For each class of CQA, the content of the major isomer was negatively affected by cooking. For monoCQAs, the content of 5-CQA was significantly reduced at 7 and 10 min compared to the raw sprouts (Figure 2). Among the diCQAs, we observed a significant decrease in the predominant isomer (3,5-diCQA) at each time point during cooking compared to the raw sprouts, with the lowest values at 7 and 10 min (Figure 2).

### Effect of Low-Temperature Storage on the CQA Content of Sunflower Sprouts

**Figure 3:**
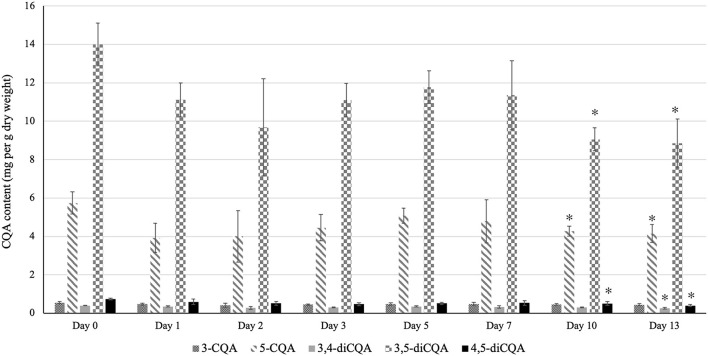
Effect of low-temperature storage on the CQA content of sunflower sprouts. The CQA profile of sunflower sprouts were analyzed after storage under simulated home-refrigeration storage conditions for 13 days. Error bars represent means ± standard deviations (SD), and three independent biological replicates were performed. For each CQA derivative, an asterisk (*) above the bars indicates a significant difference compared to the sample at day 0 (control) (Student's *t*-test, *P* < 0.05). The content of 3-CQA did not vary significantly during storage compared to the control sample (Student's *t*-test, *P* > 0.05).

Storing sunflower sprouts in a refrigerator at home and consuming as desired is a regular practice among consumers. Therefore, to evaluate the effect of storage period on the CQA profile of sunflower sprouts, we analyzed the CQA content of sprouts after storage under simulated home-refrigeration storage conditions for 13 days. As shown in Figure 3, the total CQA content of sunflower sprouts remained unchanged until day 7 post-storage. However, on days 10 and 13, the total CQA content declined as a result of the significant decrease in the content of major isoforms (5-CQA and 3,5-diCQA), which reached their lowest values at days 10 and 13 post-storage (Figure 3).

## Discussion

Plants have been commonly used as food for centuries. A large body of scientific evidence has suggested a strong association between a high intake of plant-based food products and reduced risk factors for major chronic diseases in humans, such as cancer, CVD, and age-related degeneration. These health-promoting properties are attributed to their rich content of bioactive compounds that possess antioxidant activity, including CQAs. Previous studies have documented the health-promoting characteristics of CQAs, including the antioxidant, anti-inflammatory, anticancer, antidiabetic, antihypertensive, and anti-neurodegenerative properties.

In view of the ever-increasing interest in healthy lifestyles among health-conscious consumers, sprouts are recognized as wellness- and health-promoting foods, widely recommended by dietitians, owing to their high content of bioactive compounds, including CQAs (24, 25). Apart from their health-promoting properties, sprouts are considered innovative and popular culinary ingredients because of their delicate texture, high palatability, and unique color.

Herein, we profiled the CQA content of some sprout species commonly found on the market, including pea, white radish, peanut, sunflower, mung bean, broccoli, Chinese kale, purple cabbage, mustard, soybean, and green pea sprouts, out of which sunflower sprouts harbored the highest total CQA content at around 22 mg/g dry weight. Despite the numerous health benefits of CQAs in humans, data on their content and distribution in plants and foods are limited. In addition, most of the existing data include the content of 5-monoCQA (chlorogenic acid), which is the most abundant CQA in nature. Of particular note, the chlorogenic acid content of sunflower sprouts (~6.7 mg/g dry weight) is greatly higher when compared to those of other plants reported in the literature, including common buckwheat sprouts (~0.27 mg/g) (47), red radish sprouts (~0.1 mg/g), mizuna sprouts (~0.5 mg/g), Chinese cabbage sprouts (~0.4 mg/g), turnip sprouts (~0.2 mg/g) (48), and red cabbage sprouts (~0.4 mg/g) (49) on a dry weight basis. Although the total CQA content of sunflower sprouts (~22 mg/g) was still lower than that of the green coffee bean (41 mg/g), it is still comparable to that of roasted coffee beans [light: 19 mg/g, medium: 10 mg/g, and dark roasted one: 5 mg/g; (50)]. Considering the easy and quick production (~5–7 days after germination) of sunflower sprouts along with their significant nutritional and culinary benefits, this sprout species deserves attention regarding the pharmacological properties attributed to its rich CQA content.

It should be noted that although the CQA content of sunflower sprouts is significantly higher than that of other sprout species reported in the current and previous studies, it should not be concluded that the lower contents observed in some of the investigated plants are not important to humans, owing to the fact that the metabolism and requirements of these compounds seem to vary among individuals, with no dietary recommendations for them.

Among the CQA isomers found in sunflower sprouts, 5-monoCQA and 3,5-diCQA were the most abundant (Table 1). Our findings are partially in line with those of previous studies by Sun et al. (28) and Cheevarungnapakul et al. (29), who reported a rich abundance of 5-CQA in sunflower sprouts. However, these studies reported 1,5-diCQA as the predominant diCQA isomer. The inconsistency between our results and those reported previously is likely due to the mis-annotation of 1,5- and 3,5-diCQA in previous reports. Of particular note, the precise identification of individual CQAs by means of chromatographic analysis is challenging due to the difficulty in distinguishing between the positional isomers, especially when they are present at low concentrations. Hence, for more accurate peak identification, the retention times and UV spectra were compared with those of the corresponding green coffee bean extract [known sample (51)]. Accordingly, we found that the peak identity was 3,5-diCQA, not 1,5-diCQA.

Sprouts are consumed in many ways. They can be added as raw ingredients to sandwiches, salads, soups, and desserts, and/or they can be cooked. Cooking may enhance the palatability of sprouts by softening the tissues, inactivating toxic and anti-nutritional compounds and microorganisms, and forming color and flavor compounds (52). In addition, cooking may soften vegetable tissues, facilitating the extraction of phenolic compounds from the cellular matrix (34). However, cooking might have adverse effects on the antioxidant capacity and nutritional quality of sprouts. Hence, it is of great interest to consumers and food industries to gain a better understanding of the effect of cooking on the content of bioactive compounds in sprouts. We assessed the effect of cooking on the CQA profile of sunflower sprouts by comparing the CQA content of cooked sprouts (steamed for 1, 5, 7, and 10 min) to that of raw (uncooked) samples. Notably, cooking had adverse effects on the total CQA content of sunflower sprouts relative to the raw (uncooked) sprouts in a time-dependent manner. The total CQA content reached its lowest value at 7 and 10 min of cooking (Figure 2). The decrease in CQA content, especially after a longer cooking period, could likely occur as CQAs are heat sensitive. The decline in CQA content as a result of heating has previously been reported for roasted coffee beans (50) and yerba mate (53) when compared to the CQA profile of green beans. Comparing mono and diCQAs, we observed that diCQAs are more heat liable than monoCQAs (Figure 2) The higher stability of monoCQAs under heat might be due to the fact that the ester bond linkage in the quinic acid moiety is less stable in an axial bond configuration than in an equatorial bond configuration (54, 55). Notably, a significant decline in the content of the predominant isomer (3,5-diCQA) was observed in cooked sprouts relative to the raw samples, with the lowest values at 7 and 10 min (Figure 2). 3,5-diCQA synthesis was found to be optimal at pH 6, which would likely take place in an acidic compartment of the cell, such as the vacuole (56). The changes in pH led to the instability of the 3,5-diCQA structure (56). The significant decrease in 3,5-diCQA content as a result of cooking might be due to the detrimental effect of heat shock on the cells, which could negatively affect not only the cell membrane but also the vacuolar membrane as a possible cellular site for injury, leading to changes in pH and instability of 3,5-diCQA, which could then be degraded and/or converted to other CQA isomers.

Of particular note, considering the effect of cooking methods and periods on the antioxidant capacity and content of bioactive compounds, there is no consensus in the literature regarding the best way to consume vegetables because of the inconsistent results obtained from different studies. Some studies have documented increases, while others have reported decreases in bioactive compound levels and antioxidant activity after cooking (42, 45, 57–59).

Low-temperature storage of sprouts at home and consumption as desired is a common practice among consumers. In our study, we investigated the effect of low-temperature storage on the CQA profile of sunflower sprouts by profiling the CQA content of sprouts after storage under simulated home-refrigeration storage conditions for 13 days. Notably, we did not observe any significant changes in the CQA content of sprouts until day 7 post-storage (Figure 3). However, on days 10 and 13, a significant decrease in the content of major isoforms (5-CQA and 3,5-diCQA) was observed, leading to a decline in the total CQA content (Figure 3) which might likely occur as a result of cell injury under cold shock. Similar to the effect of cooking, previous studies have reported different patterns of variations in the phytonutrient content of various fruits and vegetables under simulated home-refrigeration storage conditions, depending on the species and the compounds under investigation (31–39). To the best of our knowledge, our study is the first to report on the effect of low-temperature storage on the CQA content of sunflower sprouts. It should be noted that in the present study, we used targeted metabolomics to quantify the CQA content of sunflower sprouts under different conditions since CQAs are the predominant phenolic compounds in sunflower sprout with numerous health-beneficial properties. This point justifies the application of targeted metabolite profiling in our study. However, we do not mean to overlook other potential metabolites which might contribute to the nutritional quality of sunflower sprouts. Using non-targeted metabolomics to profile the bioactive compound content of sunflower sprouts under different conditions could be the subject of further investigation.

In summary, we investigated the effect of variety, cooking, and low-temperature storage on the CQA content of sunflower sprouts. All measured parameters significantly affected the CQA profiles of sprouts. Considering the numerous health-promoting benefits of CQAs, our findings provide consumers and food scientists with an improved understanding of the effect of cooking and home refrigeration on the CQA content of sunflower sprouts.

## Data Availability Statement

The original contributions presented in the study are included in the article/Supplementary Material, further inquiries can be directed to the corresponding author.

## Author Contributions

SS conceived the study. KC prepared the samples and performed the CQA analysis. GK analyzed the data. SS and GK drafted the manuscript. PB helped in optimizing the CQA analysis conditions. All authors read and approved the final manuscript.

## Funding

This work was supported by the 90th Anniversary of Chulalongkorn University [Ratchadaphisek Somphot Endowment Fund], Chulalongkorn University [GRU 6407023008-1; to SS], and Agricultural Research Development Agency, Thailand [CRP6405031890; to SS].

## Conflict of Interest

The authors declare that the research was conducted in the absence of any commercial or financial relationships that could be construed as a potential conflict of interest.

## Publisher's Note

All claims expressed in this article are solely those of the authors and do not necessarily represent those of their affiliated organizations, or those of the publisher, the editors and the reviewers. Any product that may be evaluated in this article, or claim that may be made by its manufacturer, is not guaranteed or endorsed by the publisher.
