# Supplementary material for: Sprout Caffeoylquinic Acid Profiles as Affected by Variety, Cooking, and Storage
Source: Front Nutr. 2021 Dec 13;8:748001. doi: 10.3389/fnut.2021.748001 (PMC8710737; doi:10.3389/fnut.2021.748001)
Supplement: Supplementary file 1 [file Data_Sheet_1.docx]

Supplementary Material

**
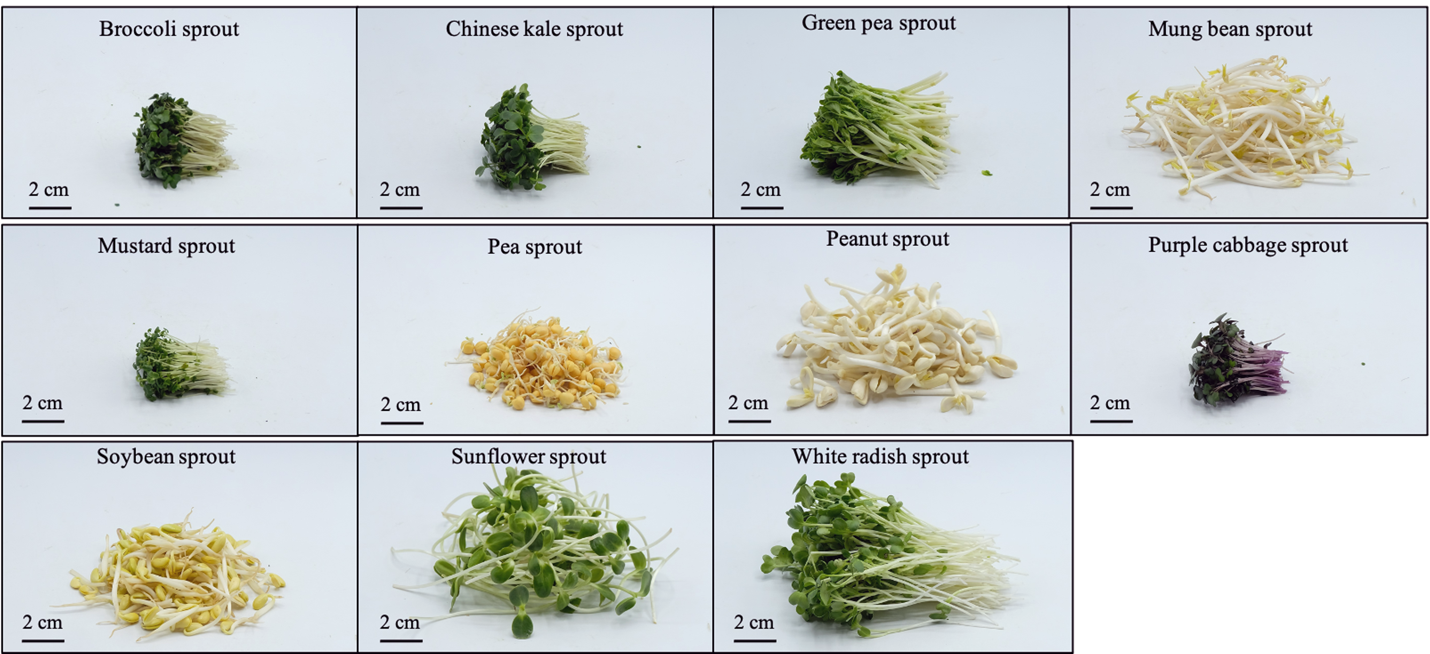
**

**Supplementary Fig. S1.** Different sprout species used in this study. Different species of sprouts commonly found in Thai local markets were used for caffeoylquinic acid (CQA) profiling. Pictures of representative sprouts are presented.

**
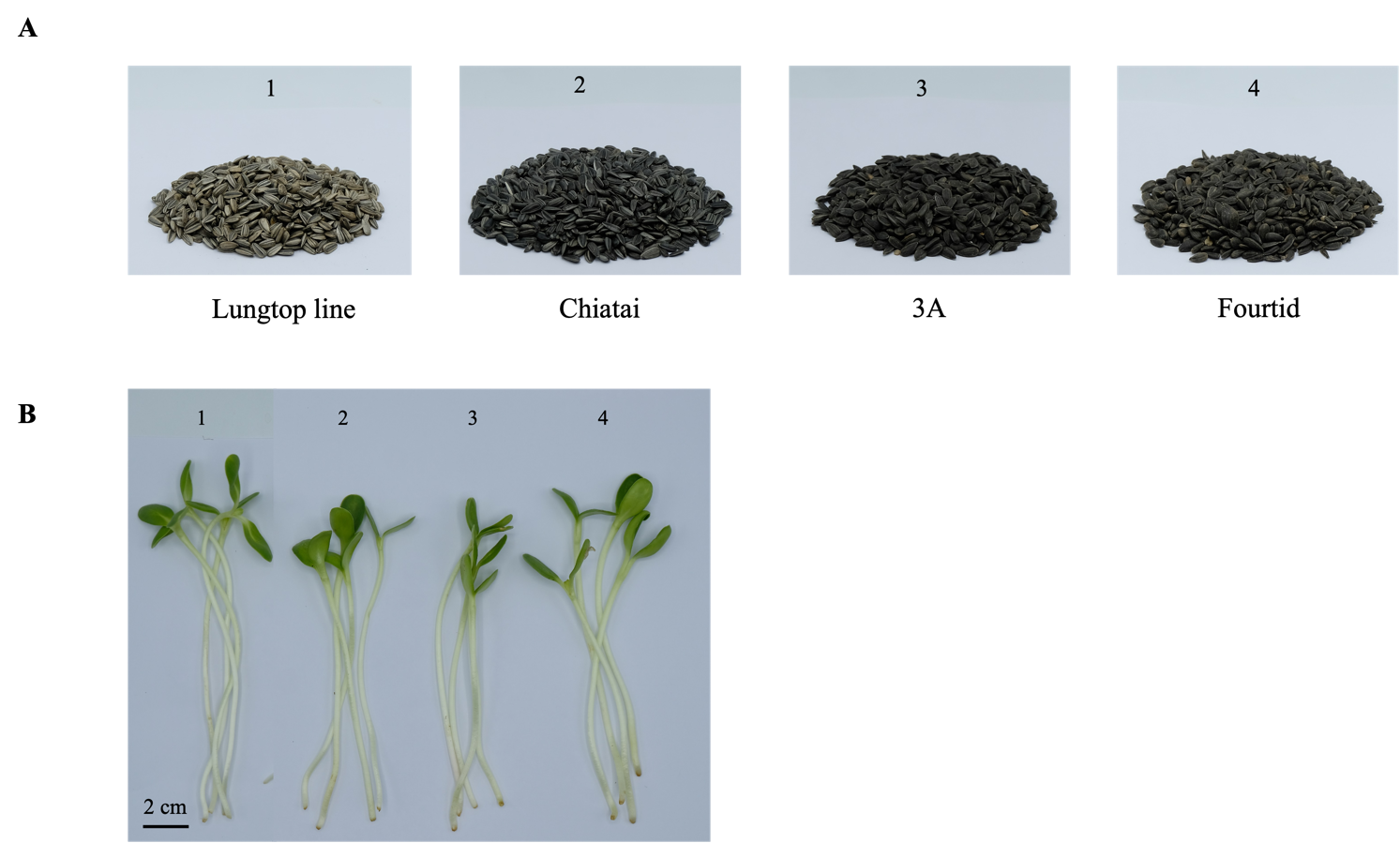
**

**Supplementary Fig. S2.** Seeds and five-day-old sprouts of four different sunflower varieties. Pictures of representative **(A)** seeds and **(B)** sprouts of the four different sunflower varieties are presented.

**
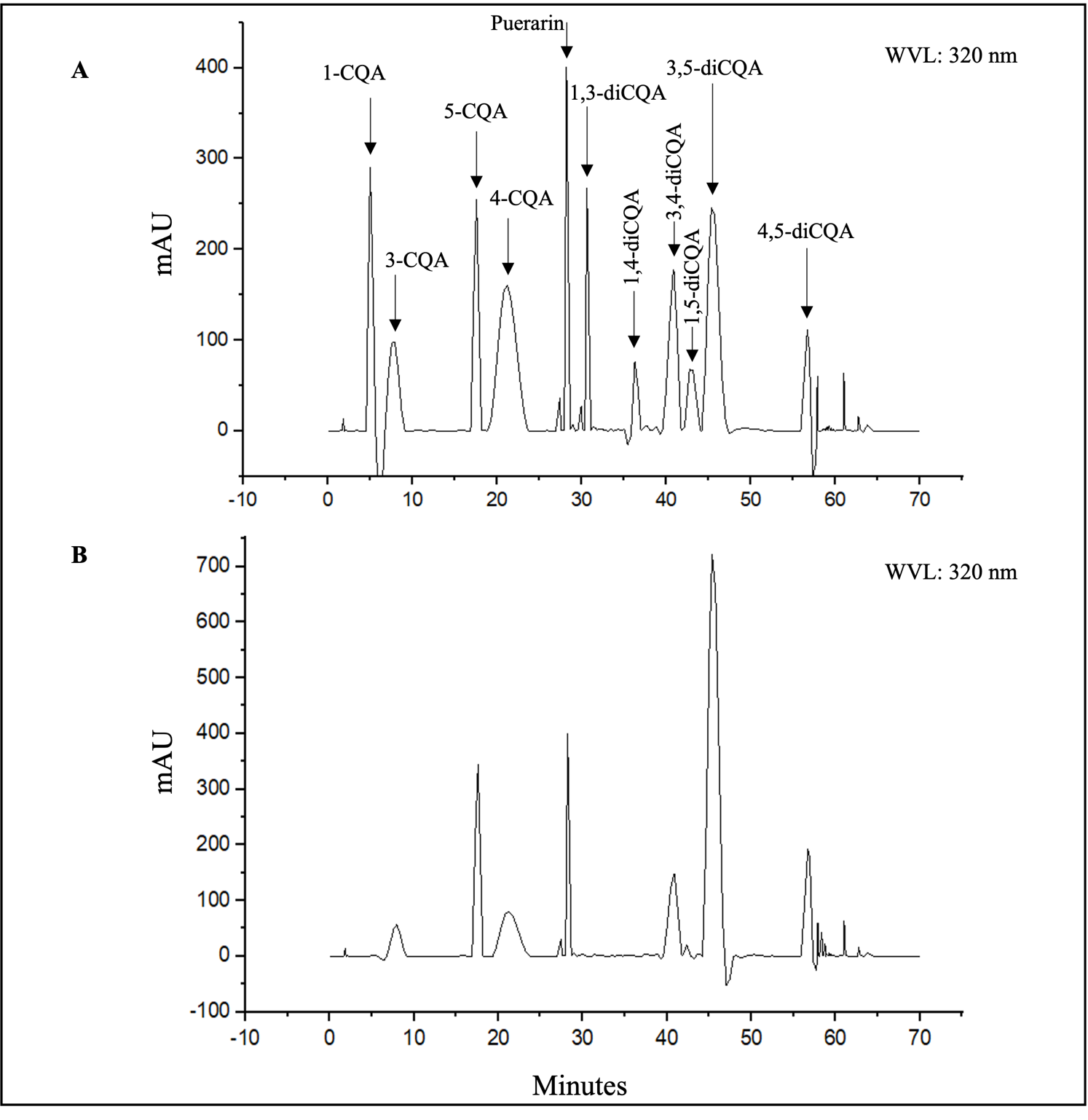
**

**Supplementary Fig. S3.** Chromatograms of the analytical standards (A) and the sunflower sprout sample (B).


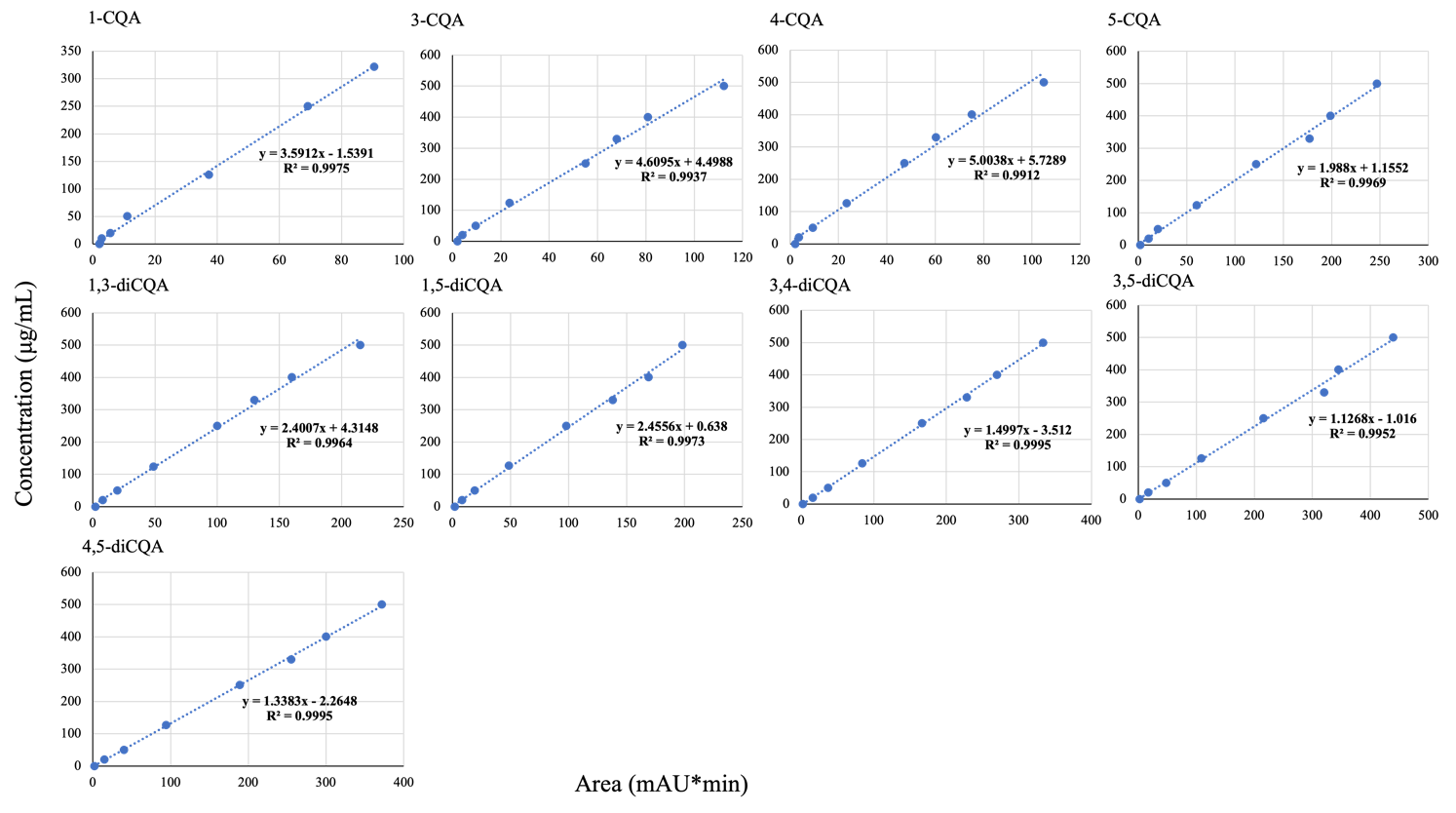


**Supplementary Fig. S4.** Standard curves and equations of standards used in this study.

**Supplementary Table S1.** The limit of detection (LOD) and limit of quantification (LOQ) of standards used in this study

| Standard | Limit of detection (LOD) (μg/mL) | Limit of quantification (LOQ) (μg/mL) |
| --- | --- | --- |
| 1-CQA | 1.09 | 3.89 |
| 3-CQA | 6.34 | 19.23 |
| 4-CQA | 6.93 | 21.00 |
| 5-CQA | 10.53 | 31.93 |
| 1,3-diCQA | 9.26 | 28.07 |
| 1,4-diCQA | 8.06 | 26.77 |
| 1,5-diCQA | 7.77 | 23.56 |
| 3,4-diCQA | 5.45 | 16.51 |
| 3,5-diCQA | 16.79 | 50.89 |
| 4,5-diCQA | 6.11 | 18.52 |
